# Administration of Adipose Tissue Derived Stem Cells before the Onset of the Disease Lowers the Levels of Inflammatory Cytokines IL-1 and IL-6 in the Rat Model of Necrotizing Enterocolitis

**DOI:** 10.3390/ijms252011052

**Published:** 2024-10-15

**Authors:** Marek Wolski, Tomasz Ciesielski, Kasper Buczma, Łukasz Fus, Agnieszka Girstun, Joanna Trzcińska-Danielewicz, Agnieszka Cudnoch-Jędrzejewska

**Affiliations:** 1 Department of Pediatric Surgery, Medical University of Warsaw, Zwirki i Wigury 63a, 02-091 Warsaw, Poland; 2 Chair and Department of Experimental and Clinical Physiology, Laboratory of Centre for Preclinical Research, Medical University of Warsaw, Banacha 1B, 02-097 Warsaw, Poland; tomasz.ciesielski@wum.edu.pl (T.C.); kasper.buczma@wum.edu.pl (K.B.); agnieszka.cudnoch-jedrzejewska@wum.edu.pl (A.C.-J.); 3 Department of Pathology, Medical University of Warsaw, Pawinskiego 7, 02-106 Warsaw, Poland; lukaszpiotrfus@gmail.com; 4 Department of Molecular Biology, Institute of Biochemistry, Faculty of Biology, University of Warsaw, Ilji Miecznikowa 1, 02-096 Warsaw, Poland; a.girstun@uw.edu.pl (A.G.); j.trzcinska-da@uw.edu.pl (J.T.-D.)

**Keywords:** necrotizing enterocolitis, inflammation mediators, adipose tissue-derived stem cells

## Abstract

There is little research concerning the role of stem cells in necrotizing enterocolitis (NEC). Bone marrow-derived mesenchymal stem cells (BMDSC) and amniotic fluid-derived stem cells significantly reduced the amount and severity of NEC in the animal models. ADSCs share similar surface markers and differentiation potential with BMDSCs. Their potential role in the setting of NEC has not been researched before. The hypothesis of the study was that prophylactic intraperitoneal administration of ADSCs before the onset of the disease will result in limiting the inflammatory response, effecting a lower incidence of NEC. On a molecular level, this should result in lowering the levels of inflammatory cytokines IL-1 and IL-6. The local ethical committee for animal experiments approval was acquired (WAW2/093/2021). We utilized a self-modified rat NEC model based on single exposure to hypothermia, hypoxia, and formula feeding. One hundred and twenty-eight rat puppies were divided into two groups—prophylaxis (ADSC-NEC, n = 66) and control group (NEC-PLCB, n = 62)—to measure the influence of ADSCs administration on the inflammatory changes in NEC, the level of cell engraftment, and the histopathology of the disease. The analysis did not show a significant effect on histopathology between groups, H(2) = 2.12; *p* = 0.347; η²H = 0.00. The intensity of the NEC variable results was similar across the analyzed groups (NEC-PLCB and ADSC-NEC). For IL-1 and IL-6, the difference between the NEC-PLCB group and the ADSC-NEC group was statistically significant, *p* = 0.002 and *p* < 0.001, respectively. To conclude, administration of adipose tissue-derived stem cells before the onset of the disease lowers the levels of inflammatory cytokines IL-1 and IL-6 but does not affect the histopathological results in the rat model of NEC.

## 1. Introduction

### 1.1. NEC, Necrotizing Enterocolitis

Necrotizing enterocolitis (NEC) is a severe neonatal condition characterized by widespread inflammation and necrosis of the bowel wall [1]. It predominantly affects premature infants, with the risk being inversely proportional to birth weight. Infants with birth weights under 1000 g experience the highest morbidity and mortality rates. NEC’s incidence is estimated at approximately 1 in 1000 newborns, rising to 20% in those with very low birth weight (VLBW) [2]. Mortality rates range between 15% and 30%, with gestational age and birth weight being key survival predictors [2]. Despite NEC being a major indication for neonatal surgical intervention, the underlying pathophysiology remains largely unknown. Hypoxia, reduced intestinal perfusion, and bacterial colonization are frequently cited as contributory factors [3]. Risk factors include prematurity, low birth weight, early formula feeding, and intestinal dysbiosis. Additionally, maternal conditions such as infections during pregnancy, metabolic disorders, substance abuse, and hypoxia are considered potential contributors [1,2]. The management of NEC varies from medical to surgical interventions, with bowel perforation mandating surgical exploration. Conservative approaches involve broad-spectrum antibiotics, cessation of enteral feeding, and gastrointestinal decompression [2,4]. Surgical strategies depend on the extent of necrosis and the infant’s clinical status. In terms of prevention, breast milk feeding is the most strongly recommended practice, while the role of probiotics remains under debate [1]. NEC research commonly utilizes animal models, including rats, mice, and pigs. Rat models are essential for testing therapeutic efficacy, while genetically modified mouse models provide further insights into the disease’s molecular mechanisms [5,6]. Piglet models, although costly, offer the closest anatomical and physiological similarities to the human gastrointestinal system [5].

### 1.2. Mesenchymal Stem Cells

Stem cells (SCs) are unspecialized cells capable of self-renewal and differentiation into various cell types [7]. Of the two primary categories—embryonic and adult stem cells—the latter are preferred in research due to fewer ethical concerns. Mesenchymal stem cells (MSCs) have shown promise in regenerative medicine due to their ability to proliferate, differentiate, modulate immune responses, and reduce inflammation. This makes MSCs an attractive candidate for therapeutic applications in conditions like NEC.

### 1.3. Cytokines in NEC

The cytokine pathway plays a central role in the pathogenesis of necrotizing enterocolitis [8]. Initial damage caused by hypoxia and hypothermia results in intestinal mucosal damage, which combined with formula feeding and bacterial colonization of the immature mucosa results in production of several cytokines such as platelet activating factor (PAF) and tumor necrosis factor alpha (TNF-alpha). Bacterial products such as lipopolysaccharide (LPS) also weaken the regenerative potential of the mucosa by inhibiting enterocyte migration [8,9]. Resulting in the leak in mucosa, this process leads to bacterial migration, vasoconstriction, an inflammatory cascade, and finally ischemia and intestinal wall necrosis [8,9]. Human NEC specimens show increased mRNA expression of interleukin (IL)-1B, IL-8/chemokine CXC-motif ligand (CXCL)-8, and tumor necrosis factor (TNF) [10]. MohanKumar et. al. analyzed microarray data from NEC bowel samples and normal intestine. NEC increased the expression of many cytokines, e.g., IL-1A, IL-1B, IL-6, IL-10, TNF, hepatocyte growth factor (HGF), and vascular endothelial growth factor (VEGF)-A [10]. Also, plasma levels of cytokines like IL-6, IL-8/CXCL8, and IL-10, among others, are elevated during necrotizing enterocolitis [10].

Interleukin-1, released from macrophages, has two components: IL-1alpha, a cell-associated protein, and IL-1beta, the secretory molecule. Stimulated by TNF-alpha, it promotes the inflammatory response. It is connected to the onset of fever, increased endothelial leukocyte adhesion, phagocyte activation, and lymphocyte stimulation. Interleukin-1beta has been shown to increase the neutrophil population at the inflammation site by activation of the IL-8 gene. IL-1 plays a dominating role in the systemic inflammatory response syndrome (SIRS), which can lead to sepsis and multisystem organ failure [8].

The release of interleukin-6 is upregulated by many other proinflammatory cytokines, e.g., TNF-alpha and IL-1 [8,10]. It activates lymphocytes and induces antibody secretion by B cells and cytotoxic T-cell differentiation [8,9]. During SIRS and sepsis, there is an increase in the concentration of enterocyte-secreted IL-6, for it is expressed by intestinal endothelium, macrophages, and helper T cells [8]. It is augmented by bacteria, endotoxins, and other cytokines [8]. Acute phase reactants like C-reactive protein are also activated by IL-6 [8].

### 1.4. Adipose-Derived Stem Cells and NEC

Stem cells can be isolated from a variety of tissues, including the liver, kidney, skin, bone marrow, adipose tissue, placenta, and umbilical cord blood [11,12]. While MSCs from different sources share similar biological properties, they exhibit differences in morphology, immunophenotype, proliferative potential, and therapeutic application [13]. Adipose-derived stem cells (ADSCs) are gaining attention as a reliable source of MSCs due to the abundance of adipose tissue and the minimally invasive nature of harvesting these cells [14]. ADSCs exhibit properties similar to bone marrow-derived MSCs (BM-MSCs), including the ability to differentiate into various cell types derived from all three germ layers [15]. Importantly, one gram of adipose tissue can yield approximately 5 × 10^9^ stem cells, significantly more than what can be obtained from bone marrow [16]. ADSCs retain their multipotency even after multiple cell passages [14]. ADSCs have been shown to secrete angiogenic growth factors, cytokines, and chemokines, all of which may have therapeutic implications in reducing the severity and incidence of NEC in animal models [17]. Preliminary studies on bone marrow-derived and amniotic fluid-derived MSCs have demonstrated significant reductions in NEC severity in animal models, and ADSCs may offer similar benefits given their comparable surface markers and differentiation capacities [12,15,18,19,20,21].

### 1.5. Immuno-Regulatory Properties of ADSCs

There are two main mechanisms by which stem cells affect the immune system—directly via cell–cell communication and indirectly through the secretion of soluble mediators, growth factors, and extravascular vesicles [22]. Immunoregulation based on direct cell–cell contact with immune cells is attributed to paracrine secretion of soluble mediators, cytokines, and growth factors [22]. ADSCs have been shown to impact different immune cells like T cells, B cells, and macrophages [23,24]. They have increased the production of de novo regulatory T cells [25,26], stimulated the polarization of macrophages into the M2 phenotype, downregulated the expression of pro-inflammatory cytokines (TNF, IL-1, IFN, and IL-12), and upregulated the secretion of the anti-inflammatory cytokines (IL-10) [27,28,29]. ADSCs secrete soluble mediators and extravascular vesicles (exosomes and microvesicles) [30,31]. The effect of modulating the immune response is mainly through paracrine mechanisms (7, 35, 71). Soluble mediators are comprised of numerous pro- and anti-inflammatory cytokines and other factors (e.g., vascular endothelial growth factor (VEGF), insulin-like growth factor (IGF-1), and granulocyte colony-stimulating factor (G-CSF). In a clinical setting, extravascular vesicles proved effective in stimulating wound healing, affecting the proliferation of human dermal fibroblasts [32]. ADSC-derived exosomes are lipid membrane nanovesicles (30–100 nm) secreted by the cells [22]. They carry a composition of proteins, lipids, DNA, mRNAs, micro-RNAs, tRNAs, and noncoding RNAs [22,33,34]. The biological properties of MSC exosomes originating from various sources are similar, as suggested by Lai et. al. [35]. ADSC exosomes exert their therapeutic effect through a paracrine mechanism by releasing their content to the cytosol of target cells. They release contents to the target cell’s cytosol in a paracrine fashion [22]. The effect of exosomes on the immunological system is believed to be like ADSCs. The exact mechanism of action is, however, unknown [22]. The clinical properties demonstrated in an experimental setting involved, among others, the impact of exosomes on wound healing by increasing the synthesis of collagen [36,37], decreasing the inflammatory cell count in the dermatitis skin [38], and reducing cardiomyocyte apoptosis caused by oxidative stress [39].

### 1.6. Aim of the Study

The hypothesis of the study was that prophylactic intraperitoneal administration of ADSCs before the onset of the disease will result in limiting the inflammatory response and promoting regeneration, thus effecting a lower incidence of NEC. On a molecular level, this should result in lowering the levels of proinflammatory cytokines IL-1 and IL-6 in the peritoneal fluid.

## 2. Results

### 2.1. Engraftment

**Figure 1:**
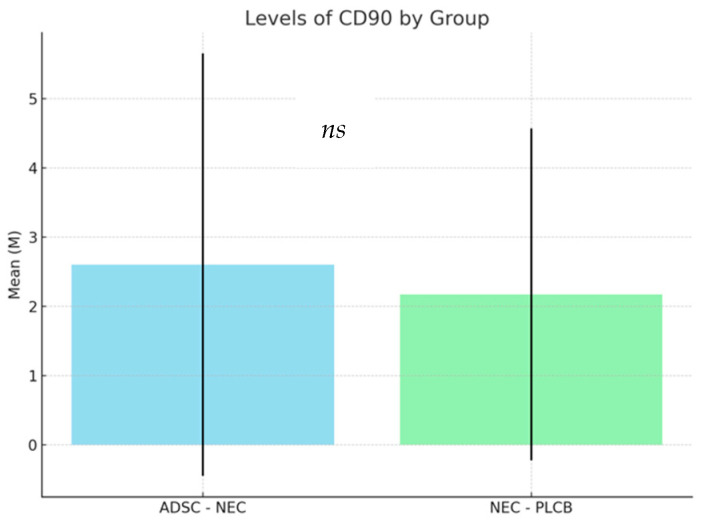
Levels of CD90 by group.

To evaluate the presence of CD90 cells in rat bowel, sections were stained with anti-CD90 polyclonal rabbit antibody (LifeSpan Biosciences, Lynnwood, WA, USA) in accordance with the manufacturer’s recommendations. Due to the non-parametric nature of the analyzed data, a Kruskal–Wallis analysis was conducted (Kruskal and Wallis, 1952). The analysis showed a significant effect of the group variable on the CD90 variable results, H(2) = 6.04; *p* = 0.049; η^2^H = 0.02. Despite the significance of the Kruskal–Wallis test, a detailed analysis of multiple pairwise comparisons performed using Dunn’s non-parametric method (Dunn, 1964) did not show any significant differences between the analyzed groups. The results of CD90 antibody levels in the ADSC-NEC and NEC-PLCB groups are presented in the Table and Figure below (Table 1, Figure 1).

### 2.2. Histopathology

To conduct the histopathological analysis, each specimen (n = 128) was fixed in a 10% neutral buffered formalin solution. Due to technical problems, we were unable to scan 6 samples from the ADSC-NEC group and 2 samples from the NEC-PLCB group. The final count of samples undergoing histopathological examination was 120. Fixed samples were embedded in paraffin, cut into 3 µm sections, and stained routinely with hematoxylin and eosin (H&E) for morphological examination.

**Figure 2:**
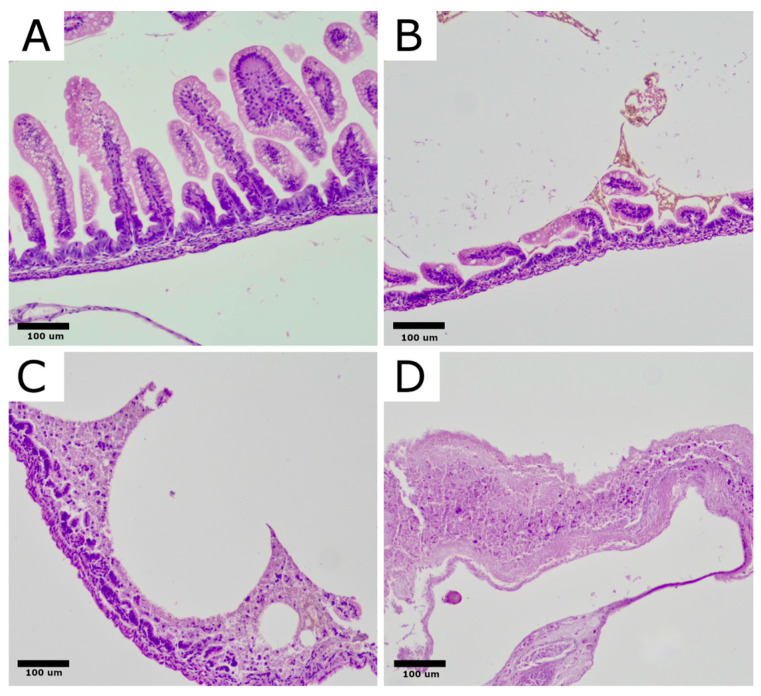
Histological changes in intestinal architecture of rats with NEC. Rat bowel stained with H&E showing representative sections for each morphological severity score. (**A**): normal ileum, NEC score 0. (**B**): NEC score 1, partial villous atrophy. (**C**): NEC score 2, sloughing and/or necrosis of upper parts of atrophic villi, (**D**): NEC score 3, total loss of villi and necrosis of the intestinal wall. Original magnification, 200×. Microphotographs were captured using OPTIKA LITEView software (Version: Windows x64 2.1.24744.20240303; OPTIKA, Ponteranica, Italy). Source: own materials.

Slides were first assessed at low magnification (40× scanning magnification) to evaluate the general architecture of rat bowel. Next, slides were viewed at 200× and 400× magnification and assessed semiquantitatively in a four-class scoring system as: grade 0: no change in the bowel wall; grade 1: partial villous atrophy; grade 2: epithelium sloughing and/or necrosis in the upper part of atrophic villi; grade 3: total loss of villi, necrosis of the intestinal wall (Figure 2).

The results of the histopathological analysis are presented in the table below (Table 2).

**Figure 3:**
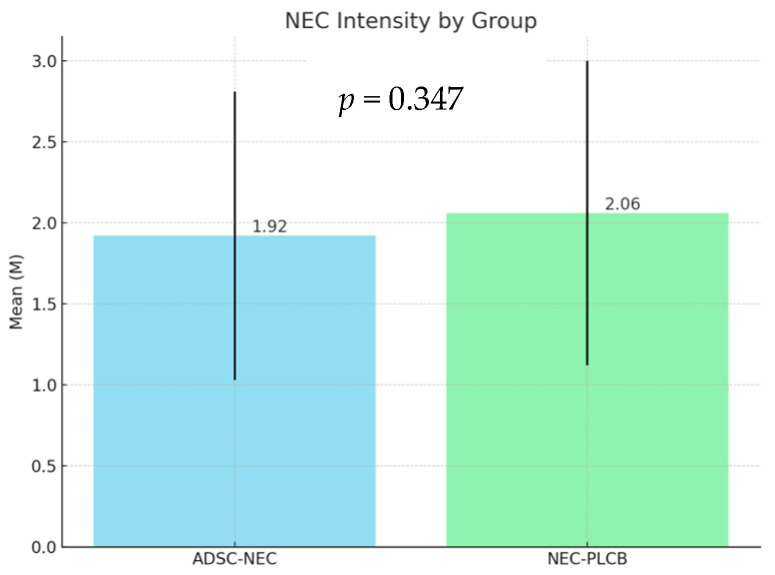
NEC intensity by group—the mean value of severity grade of all samples within each group.

Due to the non-parametric nature of the analyzed data, a Kruskal–Wallis analysis was conducted (Kruskal and Wallis, 1952) to assess the histopathological scoring data. The analysis did not show a significant effect of the group variable on the results of the NEC variable, H(2) = 2.12; *p* = 0.347; η^2^H = 0.00. The intensity of the NEC variable results was similar across the analyzed groups of the group variable. The intensity of NEC is defined as a mean of all severity scores within each of the two analyzed groups. Results are presented in the table and figure below (Table 3, Figure 3).

**Figure 4:**
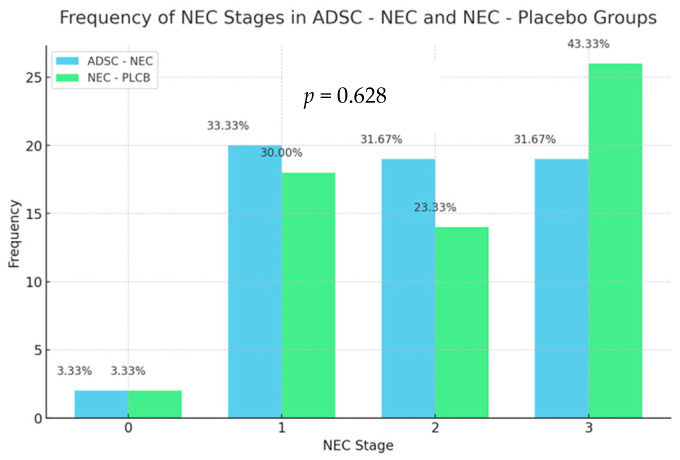
Frequency of NEC stages as defined in the grading scale (0–3) in ADSC-NEC and NEC-PLCB groups.

In order to verify the relationship between the NEC variable and the group variable, a chi-square test analysis was conducted (Pearson, 1900). Due to the presence of expected values lower than 5, a significance correction was applied using Fisher’s exact test method (Agresti, 1990; Bower and Keith, 2003). The chi-square test analysis showed no significant relationship between the NEC variable and the group variable, χ^2^(6) = 4.36; *p* = 0.628. The occurrences of the NEC variable values were similar across the different levels of the group variable. There was a tendency towards higher NEC scores in the NEC-PLCB group that did not show statistical significance. Results are presented in the figure below (Figure 4).

### 2.3. Cytokine Levels in the Peritoneal Fluid

Levels of IL-1 and IL-6 in the peritoneal fluid of ADSC-NEC and NEC-PLCB groups (n = 128) were analyzed with commercial ELISA kits. The results are presented in the tables below (Table 4 and Table 5).

**Figure 5:**
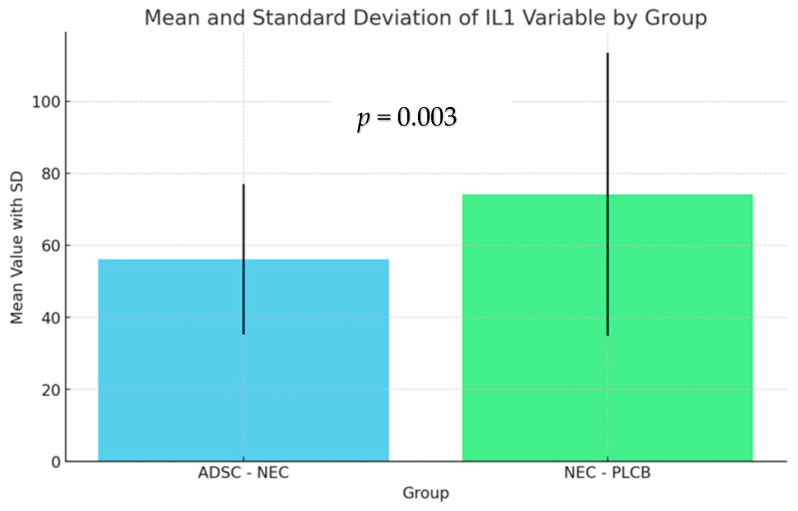
Levels of IL-1 within groups.

**Figure 6:**
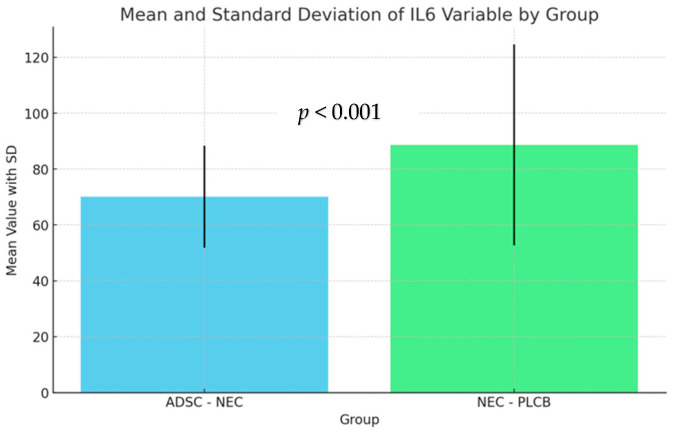
Levels of IL-6 within groups.

Levene’s test (Levene, 1960) analysis revealed that the assumption of equal variances was not met in the tested groups. Due to the unequal variances of the variables among the groups, Welch’s correction (Welch, 1951) for unequal variances was applied. The analysis showed a significant effect on the IL-1 and IL-6 variables. A detailed analysis of pairwise comparisons using Tukey’s method (Mallows and Tukey, 1991) and the effect size coefficient d-Cohen (Cohen, 1988) revealed the differences between the groups. The results are presented in the figures below (Figure 5 and Figure 6).

The difference between the NEC-PLCB and ADSC-NEC groups was statistically significant for IL-1 and IL-6 (*p* = 0.003, *p* < 0.001). Average levels of IL-1 and IL-6 were higher in the NEC-PLCB group.

### 2.4. Summary of the Results

There were no significant differences between the analyzed groups with respect to CD90 antibody levels.

The intensity of NEC was similar across the analyzed groups.

There was a tendency towards higher NEC scores in the NEC-PLCB group that did not show statistical significance.

The difference between the NEC-PLCB and ADSC-NEC groups was statistically significant for IL1 and IL6 (*p* = 0.003, *p* < 0.001). Average levels of IL-1 and IL-6 were higher in the NEC-PLCB group.

## 3. Discussion

We hypothesized that the prophylactic administration of ADSCs prior to the onset of NEC in a rat model would reduce the levels of pro-inflammatory cytokines IL-1 and IL-6, compared to the NEC group treated with saline. This result would suggest a potential future role for ADSCs in NEC therapy. NEC triggers a significant inflammatory response, with marked elevations in both mucosal and systemic cytokines, including IL-1 and IL-6, which are consistent with the disease’s pathophysiology [8,10]. In contrast, mesenchymal stem cells (MSCs) have demonstrated the ability to modulate inflammation, largely by reducing the production of inflammatory cytokines through paracrine signaling [40,41,42]. Yang et al. showed that bone marrow MSC-derived extracellular vesicles could decrease levels of inflammatory cytokines in a rat model of colonic inflammation [40]. Similarly, Ocansey et al. provided detailed evidence of MSC-mediated reductions in cytokines in models of inflammatory bowel disease [41]. To investigate this, we employed a modified version of the hypoxia–hypothermia NEC model, which has previously been used to demonstrate the prophylactic effects of maternal milk against NEC [43]. We showed a statistically significant difference between the ADSC and placebo groups in the levels of IL-1 and IL-6 in the peritoneal fluid of the NEC rats, with ADSC injection having the prophylactic potential on the intensity of inflammation. To our knowledge, this is the first time ADSCs have been tested in the context of necrotizing enterocolitis. Other types of MSCs, including amniotic fluid- and bone marrow-derived stem cells, have been studied in NEC models, showing efficacy in reducing the severity of the disease. McCulloch et al. observed reductions in NEC severity across different MSC types in rat models [20]. Similarly, Zani et al. found that AFS cells integrated into the intestinal wall and improved survival rates in rats, reducing NEC incidence and intestinal damage while enhancing enterocyte proliferation and inhibiting apoptosis [21].

ADSCs are a subset of mesenchymal stem cells found in adipose tissue, which can be easily harvested using liposuction techniques and present fewer ethical challenges compared to other stem cell sources. These cells share many characteristics with other MSCs, including their immunomodulatory, anti-inflammatory, and pro-angiogenic properties [22,44]. In the only study to date examining adipose tissue in the context of NEC, Mimatsu et al. demonstrated that differentiated adipocytes, and not ADSCs, improved mortality and promoted intestinal healing in NEC by modulating fatty acid-related proteins and reducing inflammation, including IL-1 and IL-6 levels [45].

Most experimental studies on MSCs in NEC have focused on histopathological analysis of the intestine. Previous research by McCulloh et al. demonstrated that various types of stem cells reduced the incidence and severity of NEC, as measured by histopathological assessments [18,20]. While we hypothesized that reducing inflammation with ADSCs would also lead to less severe histopathological changes, we did not observe significant differences between the two groups in terms of NEC severity or the frequency of advanced disease stages. This could be due to the modified NEC model we used, which involved a single exposure to NEC-inducing factors [43]. We assume that either the time to harvest of the bowel samples was too long or the stimuli caused by one period of hypoxia and hypothermia were too weak to elucidate the effect of ADSCs’ injection on the histopathology. We hypothesize that utilization of the established hypoxia–hypothermia–formula feeding model like in the approaches by McCulloch or Pisano might be more effective in showing this effect [18,20,46].

Considering the prophylactic effect of ADSCs administration before the onset of NEC in our study, one might hypothesize that the potential protective effect of MSCs might lower the incidence of NEC in patients with high risk of developing the disease and not only act as a curative agent. This effect was demonstrated in studies conducted by Pisano et al. [46]. Their research has focused on identifying the role of stem cells, heparin-binding epidermal growth factor-like growth factor, and stem cell-derived exosomes in preventing the disease [46]. Since the effects could be observed before the possible engraftment of stem cells into the bowel tissue, they hypothesized that the major effect of stem cell administration in NEC prevention is not engraftment but paracrine or endocrine secretion of factors, exosomes, or vesicles [46]. This is consistent with our result of the low engraftment level of the cells shown by the lack of significance in the difference between CD90 molecule levels between groups. In a separate arm of this research, we were able to show significantly different levels of the CD90 molecule when ADSCs were injected later into the disease development when the bowel walls are changed by inflammation.

The use of MSCs in neonatal medicine, particularly in the treatment of diseases affecting the brain, heart, lungs, and intestines, has gained significant scientific attention in recent years [47]. One clinical case of umbilical cord-derived MSCs being used to treat NEC was reported by Akduman et al., where a single dose of allogeneic umbilical cord mesenchymal stem cells (UCMSCs) was administered to an infant after bowel resection due to NEC, with favorable clinical outcomes [48]. Although this case study was not designed to evaluate the direct effects of MSCs, with effects measured with ultrasonography and the timing of oral feeds, it highlights the safety of MSC use in a clinical setting [48].

The limitations of this study include the arbitrary timing of ADSC administration. Future studies should explore the effects of administering ADSCs at different points during NEC development to assess whether earlier intervention could yield more significant histopathological or clinical improvements. Additionally, while we observed a trend towards reduced cytokine levels, this did not translate into clear histopathological differences. Our model, previously used to investigate the prophylactic effects of maternal milk on NEC incidence [43], may require refinement to better demonstrate the curative potential of stem cells. Other models, such as those used by McCulloh, may be more effective in demonstrating histopathological changes following MSC treatment [18,20]. Also, another study is designed to demonstrate the effect of ADSC administered after the onset of NEC to measure the impact of curative potential in the context of inflammation control.

## 4. Materials and Methods

Local ethical committee for animal experiments approval was acquired (WAW2/093/2021). We used an own modification of a hypoxia, hypothermia, and formula feeding rat NEC model to demonstrate the effect of intraperitoneal administration of ADSCs before the onset of the disease on the inflammatory profile and histopathological picture of NEC [43].

Sixty-six newborn SPRD rat puppies in the experimental group (ADSC-NEC) were subjected to intraperitoneal injection of 6 × 10^5^ labeled cells in 50 μL PBS each. Sixty-two animals in the placebo group (NEC-PLCB) were injected with 50 μL of PBS each.

The calculation of the required sample size corresponds to the number needed to demonstrate the primary endpoint of the study. The assumptions were based on the results of a pilot study and the literature [43]. The primary endpoint of the study is the percentage of animals that develop NEC in a given group, with a planned comparison of the experimental group relative to the control group. According to the adopted assumptions, the expected percentage of NEC occurrences in the experimental group is about 30 percentage points lower than in the control group, and the expected percentage of animals developing NEC in the control group is approximately 75%. Assuming that the percentages will be compared between groups using the chi-square test, with a significance level of 0.05 and a test power of 70%, the required number of animals per group is 30. Assuming a 50% dropout rate (estimated based on the pilot study results), the required number of animals per group is 60.

### 4.1. NEC Induction

The animals were subjected to the NEC protocol. They were exposed to 100% nitrogen atmosphere for 60 s in IVS cages; after one minute, normal conditions were restored. The cages were placed in a cooling device with a temperature of 4 °C for 10 min; after this time, normal conditions were restored. Animals were fed with a tolerated volume of commercially available formula milk by a 0.5 mm pipette. After a set time of 72 h (or less in case of spontaneous death but not less than 24 h) bowel fragments were obtained and analyzed.

### 4.2. Stem Cells

**Figure 7:**
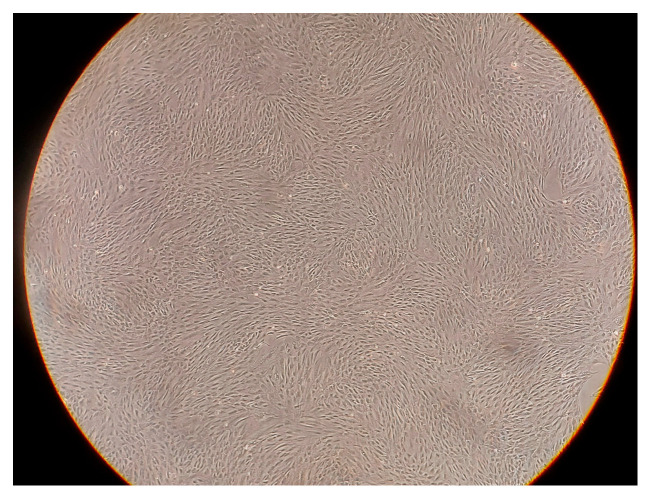
Stem cells culture. Picture captured with light microscope Olympus ck30 (Olympus optical Co., Ltd., Tokyo, Japan), WIK10×/20 L Mangnifiation 40×. Source: own materials.

StemPro human adipose-derived stem cells (Gibco, Thermo Fisher Scientific, Life Sciences Solutions, #R7788110, Carlsbad, CA, USA) were cultured in MesenPRO RS Basal medium with the addition of MesenPRO RS growth supplement (Gibco, provided with ADSC cells), 2 mM L-glutamine, streptomycin (100 μg/mL), and penicillin (100 U/mL). The cell cultures were cultivated at 37 °C in a humidified atmosphere of 5% CO_2_ (Figure 7). For experiments, cells below passage 5 were used. After reaching 80% confluence, the cells were washed with a PBS buffer and detached from vessels with Accutase^®^ solution. Subsequently, the cells were collected, centrifuged at 250× *g* for 5 min at room temperature, counted, washed with PBS, and centrifuged again. Then, cells were re-suspended in PBS and divided into portions of 6 × 10^5^ cells/50 µL.

### 4.3. Immunohistochemistry—Engraftment Analysis

**Figure 8:**
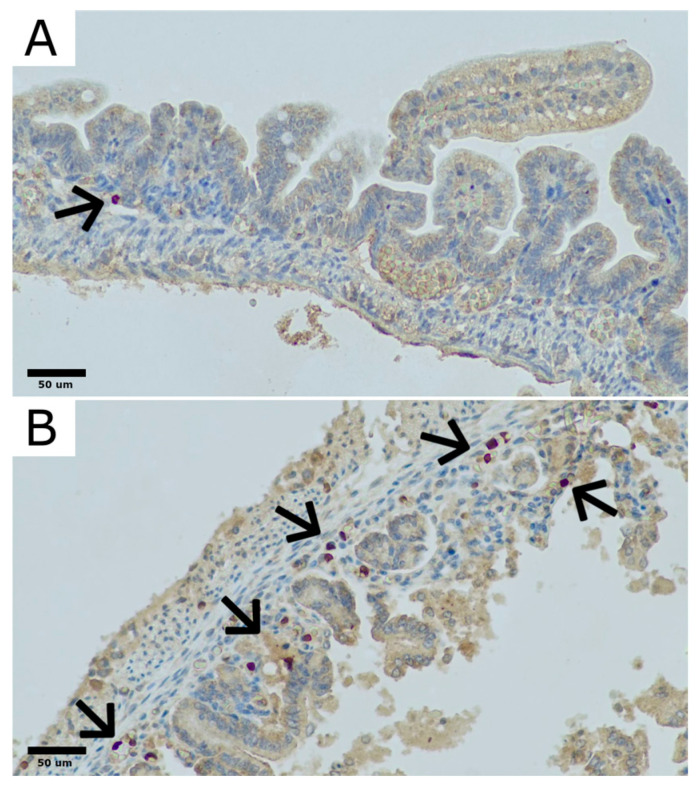
Examples of CD90-positive cells distribution in rat bowel. (**A**): Section of colon with partial villous atrophy and only a single CD90-positive cell (marked with arrow). (**B**): Section of colon with sloughing and necrosis of upper parts of atrophic villi and scattered CD90-positive cells in mucosa and submucosa (marked with arrows). Original magnification 400×. Microphotographs were captured using OPTIKA LITEView software (Version: Windows x64 2.1.24744.20240303; OPTIKA, Ponteranica, Italy). Source: own materials.

To evaluate the presence of CD90 cells in rat bowel sections were stained with anti-CD90 polyclonal rabbit antibody (LifeSpan Biosciences, Lynnwood, WA, USA) in accordance with the manufacturer’s recommendations (Figure 8). Regions with the largest number of CD90-positive cells in rat bowel were chosen at 100× magnification (hot spots), and then the number and distribution of CD90-positive cells were evaluated quantitatively in those regions at 200× magnification as a percentage of all cells present in the bowel mucosa and submucosa.

### 4.4. Histopathology

Each specimen was fixed in a 10% neutral buffered formalin solution. Fixed samples were embedded in paraffin, cut into 3 µm sections, and stained routinely with hematoxylin and eosin (H&E) for morphological examination. Slides were first assessed at low magnification (40× scanning magnification) to evaluate the general architecture of rat bowel. Next, slides were viewed at 200× and 400× magnification and assessed semiquantitatively in a four-class NEC severity scoring system as: grade 0: no change in the bowel wall; grade 1: partial villous atrophy; grade 2: epithelium sloughing and/or necrosis in upper part of atrophic villi; grade 3: total loss of villi, necrosis of the intestinal wall.

### 4.5. ELISA Analysis—Cytokine Concentrations

IL-1 and IL-6 concentrations were quantified in all collected peritoneal fluid samples using Quantikine ELISA kits (R&D Systems, Minneapolis, MN, USA) according to the enzyme-linked immunosorbent assay (ELISA) protocol. This method was applied to precisely measure IL-1 and IL-6 levels across all specimens.

## 5. Conclusions

Administration of adipose tissue-derived stem cells before the onset of the disease lowers the levels of inflammatory cytokines IL-1 and IL-6 in the rat model of necrotizing enterocolitis.

Administration of adipose tissue-derived stem cells before the onset of the disease does not significantly affect the histopathology of the disease in the rat model of necrotizing enterocolitis.

Further research is necessary on the potential prophylactic role of adipose tissue derived stem cells in the setting of necrotizing enterocolitis.

## Figures and Tables

**Table 1:** Levels of CD90 by group.

Group	n	Min	Max	M	SD
ADSC-NEC	60.00	0.00	15.00	2.60	3.05
NEC-PLCB	60.00	0.00	10.00	2.17	2.40

**Table 2:** Histopathological results. Number of samples in each severity grade.

Group	ADSC-NEC	NEC-PLCB
NEC		
0	2 (3.33%)	2 (3.33%)
1	20 (33.33%)	18 (30.00%)
2	19 (31.67%)	14 (23.33%)
3	19 (31.67%)	26 (43.33%)
Total	60 (100.00%)	60 (100.00%)

**Table 3:** NEC intensity by groups—the mean value of severity grade of all samples within each group.

Group	n	Min	Max	M	SD
ADSC-NEC	60	0	3	1.92	0.89
NEC-PLCB	60	0	3	2.06	0.94

**Table 4:** Levels of IL-1 within groups.

Group	n	Min	Max	M	SD
ADSC-NEC	66	18.45	100.32	56.10	20.85
NEC-PLCB	62	10.39	189.49	74.18	39.31

**Table 5:** Levels of IL-6 within groups.

Group	n	Min	Max	M	SD
ADSC-NEC	66	29.35	106.40	70.11	18.24
NEC-PLCB	62	25.30	180.40	88.67	35.95

## Data Availability

All data are available by request from authors.
